# Progress of Surgery in China

**Published:** 1844-09

**Authors:** 


### Collectanea.

*Progress of Surgery in China.*—Our very distinguished countryman, the Rev. Dr. Parker, Missionary Surgeon at Wampoa, has four pupils who are studying both the English language and medicine. Kwan Taon, the eldest of them, has operated successfully for cataract, upon between twenty and thirty persons; and in one instance, extirpated a tumor from a woman's shoulder, weighing, says the Missionary Herald, about a pound and a half. The patient, treated wholly by Kwan Taon, was discharged well in ten days. This young operator is represented as commanding much respect among his countrymen, and is moreover esteemed by all who know him, for his correct and gentlemanly deportment. He promises to be a useful man and a blessing to his country. It is worth remarking, that this native surgeon professes to have a higher aim than that of simply obtaining wealth—the great and prominent desire of all Chinese, in whatever capacity they are found. When he shall be qualified, according to a prescribed standard, it is presumed, of Dr. Parker's, it will be his choice, he says, to extend the benefits of the knowledge he is acquiring of foreign surgery and medicine, to other cities and other provinces of the celestial empire.

Two thousand one hundred and nine patients were admitted into the Missionary Hospital from July, 1843, to January 1, 1844. Cases of unsurpassed interest are continually presenting, and the treatment continues to be equally successful. The institution is constantly gaining upon the confidence of the Chinese of all ranks and conditions.

Yu, the late Kwang Chowfoo, being about to visit the Emperor, submitted to the removal of a tumor behind the ear, with the knife. He subsequently went to Dr. Parker's house to have the wound dressed, and once accepted an invitation to breakfast. He expressed his opinions with great freedom, discovering, by his conversation, a mind much in advance of his countrymen generally. The Imperial High Commissioner, Ke King, also availed himself of the benefits of the Missionary Hospital. On the occasion of the American Consul's presenting his credentials, at an interview with their Excellencies the Commissioners, Ke King consulted Dr. Parker in person, having done so previously by proxy.

